# The effects of salinity on nitrification using halophilic nitrifiers in a Sequencing Batch Reactor treating hypersaline wastewater

**DOI:** 10.1038/srep24825

**Published:** 2016-04-25

**Authors:** You-Wei Cui, Hong-Yu Zhang, Jie-Ran Ding, Yong-Zhen Peng

**Affiliations:** 1 Beijing University of Technology, College of Energy and Environmental Engineering, 100 Pingleyuan, Chaoyang District, Beijing 100124, China; 2 Environmental Management College of China, 8 Road of Jingang, Beidaihe District, Qinhuangdao 066102, China

## Abstract

With annual increases in the generation and use of saline wastewater, the need to avoid environmental problems such as eutrophication is critical. A previous study identified ways to start up a halophilic sludge domesticated from estuarine sediments to remove nitrogen from wastewater with a salinity of 30 g/L. This investigation expands that work to explore the impact of salinity on nitrogen removal. This study demonstrated that the mixed halophilic consortia removed nitrogen from wastewater with a salinity of 30–85 g/L. A kinetic analysis showed that halophilic nitrifiers selected based on hypersalinity were characterized by low *K*_s_, *μ*_max_ and specific ammonium oxidization rates. This explains the decrease in ammonium removal efficiency in the high salinity operational phases. Salinity inhibited ammonia oxidizing bacteria (AOB) activity, as well as the number of dominant AOB, but did not significantly affect the AOB dominant species. Three most dominant AOB lineages in the halophilic sludge were *Nitrosomonas marina, Nitrosomonas europaea,* and *Nitrosococcus mobilis. Nitrosomonas europaea* and *Nitrosococcus mobilis* were mainly affected by salinity, while nitrite accumulation and ammonia loading played the key role in determining the abundance of *Nitrosococcus mobilis* and *Nitrosococcus europaea*. The study contributes insights about shifts in halophilic nitrifying bacterial populations.

Saline and hypersaline wastewater (greater than 3% salinity) are generated from life activities in coastal cities that use seawater. Examples include seawater being used to flush toilets^1^ and seawater effluent from industries such as the tannery industry^2^, fish processing, the petroleum industry, and the leather industry^3^. Saline and hypersaline wastewaters contain high levels of inorganic salts, carbon and ammonium contaminants. These contaminants require effective treatment prior to discharging to avoid oxygen depletion and eutrophication in the receiving waters^4^. Saline wastewater makes up approximately 5% of all worldwide effluents. This proportion is expected to increase annually as industrial use expands and new saline wastewater generating industries emerge. Therefore, there is significant interest in developing efficient and economical technologies that can treat saline wastewater.

Some researchers have investigated the treatment of saline wastewater by domesticating freshwater activated sludge in saline environments^5^,^6^,^7^. These work has found that chemical oxygen demand (COD) can be removed from saline wastewater when the salinity is below 2%, and nitrogen can be removed under the salinity below 1.5%. This approach, however, has some limitations, such as unstable performance, limited salt tolerance, low pollutant removal rate, and poor settling problems^8^,^9^,^10^.

As an alternative, some studies have successfully used pure halophilic bacteria in place of their freshwater counterparts to purify organic contaminants under highly saline conditions. For example, *Halobacter halobium* ATCC 43214 achieved a high COD removal rate and efficiently treated synthetic wastewater with greater than 2% salinity^11^. *Staphylococcus xylosus* demonstrated an excellent ability to COD removal from wastewater with 7.2% salinity^5^. Using halophilic bacteria overcomes the limits associated with saline wastewater, because these bacteria survive in natural niches containing 1–30% salinity^12^.

Unfortunately, biotechnology based on pure halophilic cultures doesn’t effectively treat real wastewater in pilot-scale applications because the metabolic pathways do not adjust well to complex wastewater compositions and the shock of organic loadings. As such, mixed halophiles are needed for real large-scale hypersaline wastewater treatment. To achieve this, Sudarno *et al*.^13^ successfully obtained mixed halophilic biomass by enriching them with seawater and marine sediment samples. Most recently, a halophilic activated sludge system successfully started up and continuously operated for 140 days using inoculated estuarine sediments. This approach efficiently and stably removed total nitrogen from wastewater with 30 g/L salinity^10^.

These studies highlight the potential applications associated with biologically treating hypersaline wastewater using an engineered halophilic mixed biomass. However, treating hypersaline wastewater using mixed halophilic cultures is still poorly understood due to limited research. In particular, the community succession patterns in the halophilic activated sludge along with salinity and associated factors are important because of two reasons. First, different halophilic bacteria show significant differences in optimal salinity requirement^12^. Consequently, some halophiles with optimal salinity levels, equal to wastewater salinity, could metabolize well. Others, outside the optimal salinity range, can be inhibited or washed out from the biological community after long-term operation. As a result, community compositions vary with wastewater salinity, impacting treatment performance.

Second, wastewater salinity differs between industries and can vary between months or seasons within an industry^3^. Therefore, salinity is one of the most important parameters to be considered in real wastewater treatment plants. Other operational factors, besides salinity, can also shape the bacterial competition and community composition during treatment. There have been no reports on this factor until now. Insights on the correlation of the key operational parameters and halophilic population succession facilitate actions to optimize the treatment performance and to explain outcomes from the mixed halophilic system.

Nitrogen must be removed from hypersaline wastewater to avoid eutrophication. Nitrification is the process of biologically converting reduced inorganic nitrogen into oxidized nitrogen species. This is the first step to remove biological nitrogen. Commonly, nitrification is thought to be a rate-limiting step, and sensitive to inhibiting factors, especially salinity^14^,^15^. Compared with removing other pollutants, the biological removal of nitrogen from hypersaline wastewater is considered as the most difficult process^1^,^13^. We have previously demonstrated the feasibility of removing nitrogen from hypersaline wastewater using mixed halophilic cultures previously enriched with estuarine sediments^10^. This study laid the foundation for the further research on the nitrifier community purifying hypersaline wastewater.

This study focuses on the response of a mixed halophilic nitrifying system to salinity during a long-term experiment. Specific study objectives were to (1) investigate the effect of stepwise increased salinity ranging from 30–85 g/L on nitrification performance and kinetic, (2) establish the of mixed halophilic community succession patterns under different salinities and other operational factors, and (3) reveal the correlation between nitrification dynamics and halophilic community succession. This understanding may support new engineering approaches to using mixed halophilic nitrifying communities in wastewater treatment.

## Results

### Nitrification performance

**Figure 1:**
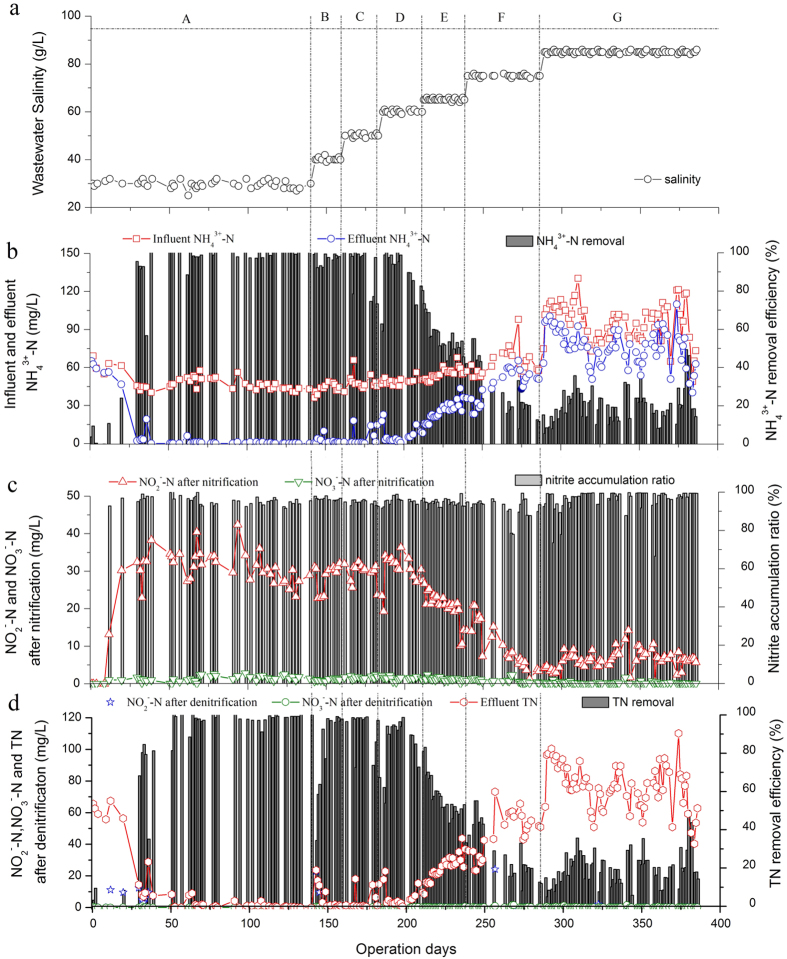
(**a**) The salinity of seven operating phases (**b**) Ammonium nitrogen removal efficiency (**c**) Nitrite and nitrate concentration after nitrification (**d**) Nitrite, nitrate and TN concentration after denitrification during operation phases. Phase A-G refers to seven operational periods treating saline wastewater with mean salinity of 30, 40, 50, 60, 65, 75 and 85 g/L.

Figure 1 shows the 400-day continuous SBR performance. The seven operating phases (A to G) were based on the influent salinity (Fig. 1a). Phase A’s influent salinity was equal to the salinity in the natural niche of estuarine (30 g NaCl/L). Estuarine sediments were seeded in phase A to enrich the halophilic bacteria. In phases B to G, salinity increased in steps at a rate of 10 g/L or less. This increase benefitted bacterial selection because it shortened the selection and acclimation time^5^,^9^. In each phase, the SBR was continuously operated over two SRTs, providing sufficient adaptation time to achieve a steady state, because treatment performance was maintained in at-least 12 continuous cycles.

Ammonium nitrogen removal efficiency exceeded 98% after 9 days and remained high in the subsequent 131 days of phase A (Fig. 1b). This suggests the successful enrichment of halophilic AOB from estuarine sediments^10^. The stable ammonium removal performance also indicated that AOB enriched from the estuarine sediment adapted to SBR operational conditions. Ammonium removal efficiency maintained stable, even when the influent salinity increased to 40 g/L in phase B and 50 g/L in phase C. However, once the salinity reached 60 g/L (phase D), ammonium was not removed completely in the first few days; it took 6 days of operation for the recovery and stabilization of complete ammonium removal.

This suggests that the increased salinity may have temporarily inhibited halophilic nitrifier activity. In phase E, ammonium removal efficiency steadily declined and reached a stable value after 15 cycles, showing significant inhibition. The trend continued during phase F, where achieving a stable ammonium removal efficiency required 20 cycles. Low and fluctuating ammonium removal efficiencies continued for all 96-day experimental period in the phase G. This result indicates that the mixed halophilic AOB in phase E, F, and G underwent selection for salinity, inhibiting the bacterial population and reducing ammonium removal.

Nitrite was the main end product of nitrification across all study phases. For phases A to D, the mean nitrite concentration was 30.516 ± 3.936 mg/L at the end of nitrification; the nitrate concentration was less than 1.685 ± 0.392 mg/L (Fig. 1c). For phase E to G, the nitrite concentration continuously decreased to 6.435 ± 2.493 mg/L at the end of nitrification due to the low ammonium oxidation efficiency; the nitrate concentration was only 0.196 ± 0.266 mg/L. Although accumulated nitrite varied as the ammonium removal efficiency changed, the nitrite accumulation ratio exceeded 95% over the experimental phases. This result indicated the second nitrification process catalyzed by nitrite oxidizing bacteria (NOB), nitrite oxidization into nitrate, almost ceased in the mixed halophilic system, allowing robust and stable nitrification.

After denitrification process, most of all the nitrite and nitrate accumulated at nitrification stage could be removed in the phases A to G (Fig. 1d). High TN removal of 92.6 ± 2.6% was achieved in the phase A to D. In the phase E to G, TN removal performance decreased to 20.9 ± 6.2% due to the high ammonium concentration in effluent, which accounted for more than 99% of the TN concentration. It could be found that the high ammonia concentration in effluent was the main factor of the decrease of TN removal in the context of elevated salinity.

### AOB community structure and evolution

**Figure 2:**
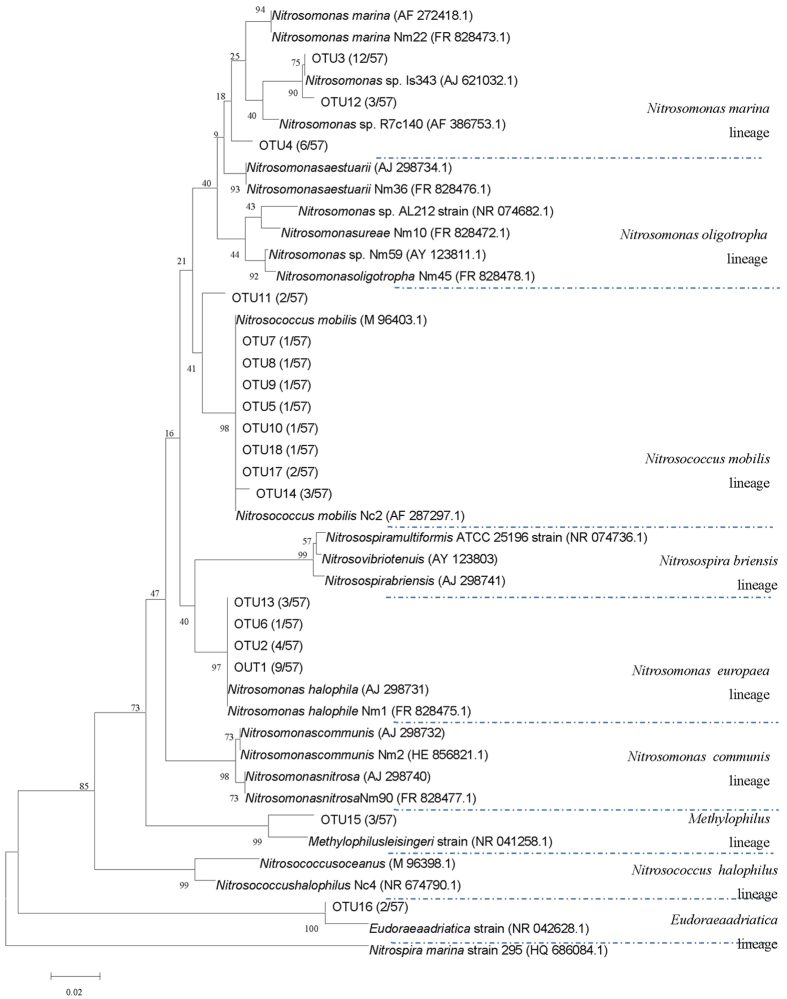
Phylogenetic relationships between AOB 16Sgene sequences. All the sequences were obtained from GenBank. The accession numbers follow the corresponding nitrifying bacteria. Numbers on the branches refer to bootstrap values; only values above 50 are shown (1000 replicates). Scale bars represent the nucleotide substitution percentage.

Figure 2 shows a AOB phylogenetic tree based on 16s rRNA sequence. Comparing the phylogenetic tree with the genes in Genbank, 57 genes were clustered to 18 operational taxonomic units (OTUs) and were further affiliated with five lineages: *Nitrosomonas marina, Nitrosomonas europaea, Nitrosococcus mobilis, Methylophilus,* and *Eudoraeaadriatica*. The *Nitrosomonas marina, Nitrosomonas europaea* and *Nitrosococcus mobilis* lineages were the three most dominant lineages, which processed 3 OTUs (21 sequences), 3 OTUs (17 sequences), and 4 OTUs (13 sequences) respectively. *Nitrosomonas marina* and *Nitrosomonas europaea* were affiliated with genera *Nitrosomonas* of *Betaproteobacteria* phylum.

The *Nitrosomonas* genus is usually found in the freshwater while *Nitrosomonas marina* and *Nitrosomonas Europe* are salt tolerant^16^. *Nitrosococcus mobilis* is affiliated with the *Gammaproteobacteria* (Holmes *et al*., 1995; Purkhold *et al*., 2000), found in marine or saline environments. No OTUs were close to the *Nitrosospira* and *Nitrosococcus halophilus* lineages although bacteria in the two lineages are often found in saline environment^16^. Both *Eudoraea adriatica* and *Methylophilus* lineages contained 1 OTUs (2 sequences and 3 sequences, respectively), not included in the AOB monophyletic lineage.

**Figure 3:**
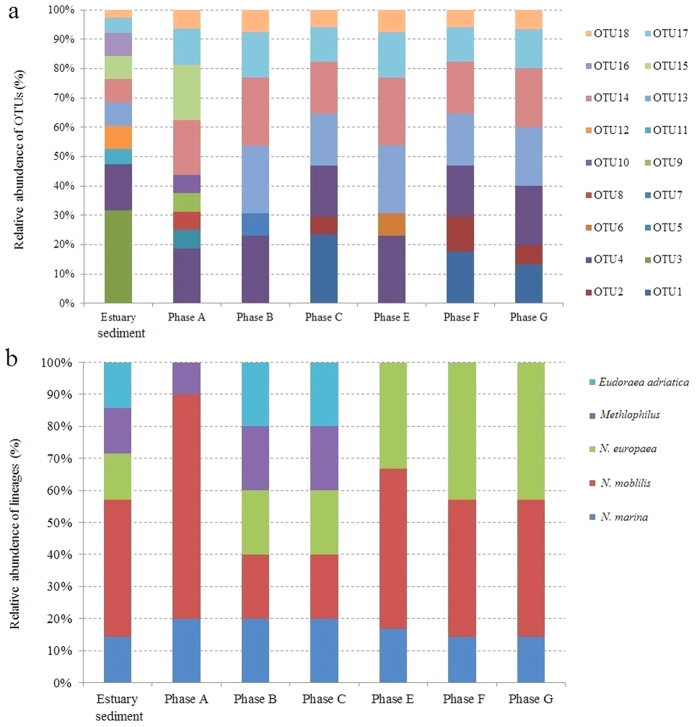
Community abundance analysis based on OTUs (**a**) and lineage (**b**).

The relative abundance based on OTUs at different operational phases were then calculated (Fig. 3a). 10 OTUs (OTU3, OTU4, OTU11-18) were detected in natural estuarine sediments. OTU3 and OTU4 belonged to *Nitrosomonas* sp. in the *Nitrosomonas marina* lineage. OTU11, OTU14, OTU17, and OTU18 were affiliated with *Nitrosococcus mobilis* in the *Nitrosococcus mobilis* lineage. OTU13 belonged to *Nitrosomonas halophile* in the *Nitrosomonas europaea* lineage.

The *N. moblilis* lineage accounted for more than 21% of the natural sediment community; *N. marina* lineage accounted for 55% of the community (Fig. 3b), making both the dominant lineages. After cultivating the seeded sediment in the SBR after 140 days in phase A, some OTUs (OTU3, OTU11, OTU12, OTU16) affiliated with *Nitrosomonas* sp., *Nitrosomonas halophile*, and the *Eudoraeaadriatica* strain disappeared. Six OTUs (OUT4, OTU13-15, OTU17, and OUT18) remained in the SBR; some OTUs (OTU5, OTU8-10) affiliated to *Nitrosococcus mobilis* were concurrently enriched.

The dominant lineage in phase A was *N. mobilis* with 59% relative abundance, derived from the sediment’s *N. marina*. The result suggested bacteria in *N. mobilis* lineage had a stronger adaptation for the conditions of artificial engineering system in contrast with *N. marina* lineage. In phase B, all the newly enriched OTUs in phase A was retained and OTU15 belonging to *Methylophilus* lineage disappeared. In phase C, OTU1 and OTU2 identified as *Nitrosomonas halophile* in *Nitrosomonas europaea* lineage were newly enriched. The dominant bacteria were shifted to *N. europaea*. In phase E, OTU6 affiliated to *Nitrosomonas europaea* lineage was detected. At the following two phases, bacterial community structure was unchanged.

Diversity analysis of bacterial community at different operation stages based on denaturing gradient gel electrophoresis (DGGE) is showed in Table 1. Shannon-Wiener, Margalef’s, and Simpson’s Diversity of estuarine sediments showed the bigger value than that in SBRs. Larger magnitude of biodiversity represented a more complex bacteria community. In Xia S. Q. *et al*.’s study^17^, the bacterial Shannon-Wiener of freshwater activated sludge was 2.341, which was same to 2.49 of the halophilic sludge in this study. The previous study concluded that salinity reduced the microbial diversity and significantly influenced the microbial community structure of freshwater activated sludge^18^. However, the decrease of Shannon-Wiener with higher salinity was not notable in our study, which indicated salinity had no significant effect on community diversity of halophilic sludge.

### NOB community analysis

The NOB community occurring in different phases and in the estuarine sediments was examined by employing probes of NIT3/EUB_mix_ and NTSPA685/EUB_mix_. No hybridization signals of NIT3/EUB_mix_ were obtained in the results of both estuarine sediments and sludge in stage A and G, implying the possible absence of *Nitrobacter sp*. (Fig. S1). There are low signal detected by NTSPA685/EUB_mix_ in the inoculated sediments, implying the low abundance of *Nitrospira mascoviensis, Nitrospira marina*, aquarium clone 710-9 in the natural estuary. However, no hybridization signals of NTSPA685/EUB_mix_ were detected in sludge in stage A and G, indicating the bacteria affliated to *Nitrospira mascoviensis, Nitrospira marina*, aquarium clone 710-9 can not survive in the engineering SBR. It can be deduced that the low abundance of NOB in the estuarine sediments prevents the halophilic NOB from being further enriched in the engineered SBR. NOB may be sensitive to environmental disturbances; there are many steep physio-chemical gradients of salinity, nitrogen, pH, oxygen, sulfide, and organic loading in the estuary^19^, which could limit NOB growth.

## Discussion

**Figure 4:**
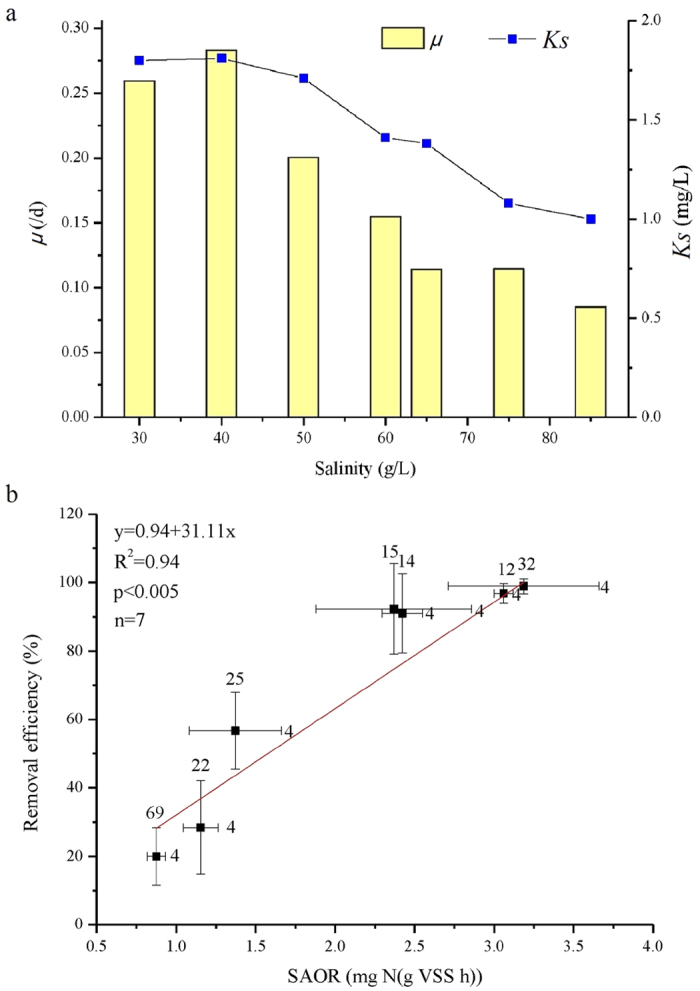
(**a**) Kinetics analysis in each operational phase. (b) The mean ammonia removal efficiency (± standard deviation) as a function of the mean specific ammonium oxidization rate (± standard deviation) in different phases as salinity increases. Numbers over bars and the right of the bars indicate sample size.

The ammonium removal efficiency varied as salinity increased (Fig. 1). Determining the reason requires establishing key kinetic parameters to furtherly characterize biological system functions. This required determining the mean specific growth rate (*μ*) and ammonium half-saturation constant (*K*_s_) (Fig. 4a). The maximum *μ* occurred at a salinity of 40 g/L. As salinity increased from 40 to 85 g/L, the mixed halophilic nitrifiers resulted in a decreased *μ*. The mixed halophilic nitrifiers sustained a high and constant *K*_s_ in phase A to C; the *K*_s_ decreased as a result of the high salinity in phases E to G. According to the Monod equation, *μ* = *μ*_max_*S*/(*S* + *K*_s_), the nitrifiers’ maximum specific growth rate (*μ*_max_) selected for high salinity decreased because of the decreased *μ*, the increased ammonium concentration (*S*), and the decreased *K*_s_ in phase E–G.

These results indicated that higher salinity selection pressure stimulated the growth of halophilic nitrifier with low *K*_s_ and *μ*_max_. *K*_s_ is an important biological factor and determines the relative affinity of a microorganism towards its substrate^20^. The selected nitrifiers, with low *K*_s_ when salinity was high, indicated strong affinity for the ammonium. Conversely, the Monod constant *K*_s_ closely depends on the state of bacterial species-substrate pairs in a microbial community^21^. Our study used a constant supply of the real municipal wastewater. As such, the change in *K*_s_ suggests that the community composition could be evolved through long-term salinity selection.

The mean specific ammonium oxidization rate (SAOR) obtained at each phase’s steady state decreased as salinity increased (Fig. 4a). Compared with phase A as a control, SAOR decreased by 25% in phase E (50 g/L salinity) and by 24% in phase F (60 g/L salinity). Significant SAOR decreases of 57%, 64% and 73% occurred in phases E, F, and G, respectively, when more than 65 g/L salinity was applied. This suggests the halophilic nitrifiers selected for high salinity resulted in the decreased SAOR. It is not surprising that the SAOR decreased, because the decreased *μ* was linked to phases with high salinity; *μ* is directly proportional to the specific substrate utilization rate (i.e., the SAOR in this study). The decreased ammonium removal efficiency related to the decreased SAOR; Fig. 4b shows the linear fit relationship. The decreased ammonium removal efficiency during high salinity phases was deduced through the decreased SAOR, because the fixed hydrogen retention time was sustained in each phase.

**Figure 5:**
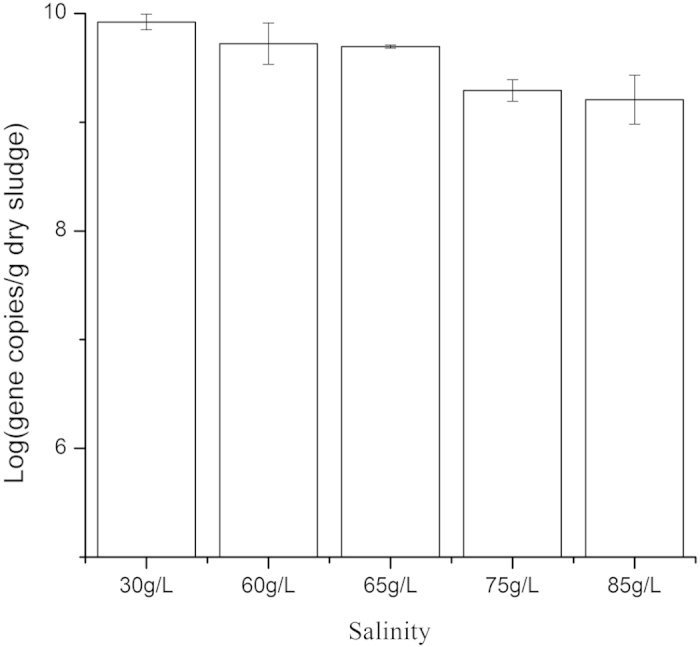
AOB 16S gene copy numbers quantified using qPCR. Error bars indicate standard deviation (n = 3).

Figure 5 shows the AOB’s 16s genes abundance of DNA extracted from the halophilic sludge. Copies/gVSS was the unit of measurement used to assess gene abundance. As salinity increased, the number of AOB declined. There were 1.5 times the number of AOB in phase A (30 g/L) than in phase D (60 g/L) and E (65 g/L). The number of AOB in phase G (85 g/L) decreased to approximately 20% of the AOB count in phase A.

Previous researchers also found that salinity was significantly negatively related to the number of AOB^22^, similar to this study. The decline in the number of AOB may result in low-rate ammonia oxidation as salinity increases. This also contributes to decreased ammonium removal efficiency from the wastewater with elevated salinity.

**Figure 6:**
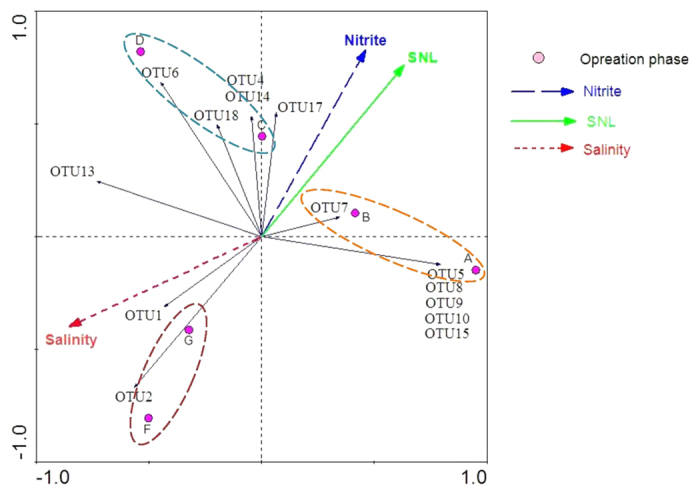
Relationship between operational phases and operational conditions to the community described using RDA analysis.

Operational parameters and community structure correlations were established using redundancy analysis (RDA) (Fig. 6). Three operational phases groups were plotted. The first group included phases A and B; the second group contained phases C and D; and the third group contained phases F and G. Further, dice coefficients (Cs) comparing PCR-DGGE fingerprint similarities also support this observation (Table S1).

Based on the operational phase clusters, three salinity ranges can be identified: low (30–40 g/L), medium (50–60 g/L), and high salinity (65–85 g/L) environments. The results are highly correlated with ammonium removal performance (Fig. 1), indicating consistency in treatment performance and community structure. The contribution of operational parameters, including of salinity, nitrite concentration, and specific NH_4_^3+^-N loading (SNL), which fluctuated across operational stages, was analyzed using RDA.

Similar to phase clusters, the OTUs were also divided into several groups in the RDA figure. The first group included OTU5, OTU8, OTU9, OTU10, and OTU15. The second included OTU4, OTU6, OTU14, OTU17, and OTU18. The third group included OTU1 and OTU2. OTU7 was positively correlated with the first and second group; OTU13 was positively correlated with the second and third group. In terms of environmental factors, salinity was positive for OTU1, OTU2, and OTU13; and negative for OTU5, OTU 7, OTU8, OTU9, OTU10, OTU15, and OTU17.

OTUs positive to salinity were affiliated to *Nitrosomonas europaea*; OTUs negative to salinity were affiliated to *Nitrosococcus mobilis*. This suggests that *Nitrosomonas europaea* can survive in hypersalinity environment, while hypersalinity poses a negative effect on *Nitrosococcus mobilis* growth. With the increase in salinity, *Nitrosomonas europaea* became the dominant species. Salt tolerance is different for bacteria, who can survive only in a certain salinity range and will not survive in environments above their salt tolerance. In our study, the fact that the number of AOB in sludge declined as salinity increased, especially when the salinity exceeds 65 g/L, can be induced by the possible disappearance of *Nitrosococcus mobilis* with low salt tolerance.

In Liu’s study^23^, the outer biofilm consisted of *Nitrosomonas* in a salinity-exposed completely autotrophic nitrogen removal over nitrite (CANON) system, supporting our research. However, species affiliated with *Bacteriodetes* and *Actinobacteria* dominated freshwater activated sludge domesticated with salinity levels of 10 g/L, 20 g/L, and 40 g/L^20^. This was a significant difference from halophilic sludge. Nitrite and NLS created similar stress, which was positive for OTU4, OTU5, OTU7, OTU8, OTU9, OTU10, OTU14, OTU15, OTU17, and OTU18; and was negative for OTU1, OTU2 and OTU13. All positive OTUs were affiliated to *Nitrosococcus mobilis*; all negative OTUs were affiliated to *Nitrosococcus europaea.* That indicated nitrite accumulation and ammonia loading played a key role in the fate of *Nitrosococcus mobilis* and *Nitrosococcus europaea*.

There are no reports explicitly describing the effect of nitrite accumulation on the halophilic AOB community, making it hard to compare this study’s results. However, a previous study did report nitrite’s inhibition of *Nitrosococcus* in freshwater activated sludge due to its toxicity^24^. As such, nitrite accumulation may have inhibited *Nitrosococcus*, changing the microorganism community structure. Because nitrite concentration was positively correlated with SNL, there was a synergistic effect with the AOB community. This effect was negative correlated with salinity. This indicates that nitrifiers can be determined by salinity in hypersaline environments, while nitrite concentration and NLS could influence the nitrifier community in low-saline environments.

Nitrification by halophilic nitrifiers makes the biological nitrogen removal from hypersaline wastewater possible. This study suggested the nitrification performance was closely related to the nitrifiers community, which presented dynamic succession with salinity. Nitrification is the most sensitive and complex biological process, influenced by many operational conditions. Further research on how other key operational parameters affect the halophilic nitrification community will contribute to insight of the halophilic microbial population succession patterns and rules in the saline environment.

## Methods

### Experimental apparatus and operational strategy

The enrichment and start-up of halophilic culture from estuarine sediments for hypersaline wastewater treatment is outlined in^10^. Sludge at 2–3 cm interface of estuarine sediments was collected from the estuary of Tang River in Qinhuangdao city of Hebei Province. The elutriation of collected sediments was carried out three times before experiment. Following this, the mixed halophilic sludge was seeded in a sequencing batch reactor (SBR). Experiments were conducted in the SBR with a working volume of 8 L, equipped with temperature control system, mechanical stirrer, and air diffusers. The SBR was fed with real municipal wastewater containing various sodium chloride salinities in seven operational phases, referred to as phases A to G. The real municipal wastewater was collected daily from a local sewer. Despite the fixed daily times of municipal wastewater deliveries, the ammonium concentrations in the wastewater still varied (Table 2). Target salinities in the municipal wastewater were synthesized by adding sodium chloride.

Table 2 lists the key operational parameters used in each phase. Compressed air was supplied at a constant rate (200 L/h) through diffusers at the bottom of the reactor. Dissolved oxygen (DO) was maintained above 3 mg/L throughout the experiment, and the temperature was kept at 24 ± 1 °C using the temperature control system. Mixing inside the reactor was performed using a mechanical stirrer.

Each complete SBR cycle consisted of 5-min feeding, 6-h aeration, 4-h anoxic mixing, 40-min settling, and 15-min decanting. At the end of aeration, 5-mL methanol was added into the SBR to provide carbon for anoxic denitrification. At the end of the reaction, some mixed liquid was discharged to control sludge retention time (SRT). During the decanting period, 5-L supernatant liquor (including the mixed liquid volume for SRT) was discharged, resulting in a volume exchange ratio of 0.625. When the ammonium removal and other biological parameters maintained stably over one SRT, the system was deemed to reach the steady state. In each phase, stable operations were maintained for at least two SRT cycles to allow for bacterial community adaptation.

All SBR operations were automatically controlled using a programmable logic controller. Influent and effluent samples were collected during each cycle to evaluate nitrification and nitrogen removal performance. After achieving stable treatment performance and biomass concentration, a mixed liquid sample was collected to determine the dynamic parameters at fixed time intervals of one cycle (one or half hour). Molecular bacterial analyses were done at the end of each phase. Table 2 also lists the specific sampling days.

### Analytical methods

DO, temperature and pH were measured using an online WTW multi 340i meter (WTW Company, Germany). Salinity was measured using a salinity meter (GMK510, Korea). NH_4_^+^-N, NO_2_^−^-N, NO_3_^−^-N, Total Nitrogen (TN), mixed liquor suspended solids (MLSS) and mixed liquor volatile suspended solids (MLVSS) were measured using standard methods^25^.

### DNA extraction, PCR amplification, and DGGE analysis

Samples for DNA extraction were collected from the reactor at the end of each phase. Approximately 0.07–0.20 g of freeze-dried activated sludge was introduced into the DNA extraction kit tubes; DNA extraction was conducted in duplicate using the Fast Soil DNA isolation kit (Qbiogene, Carlsbad, CA), using the manufacturer’s protocol. The extracted DNA was eluted with 75 uL of DES (D Nase/pyrogen-free water) and stored at −20 °C until further use.

Variable region 3 (V3) of the 16S rDNA gene was amplified using polymerase chain reaction (PCR) for ammonia oxidizing bacteria (AOB). In the amplification, approximately 50 ng genomic DNA extracted from the sludge was used as template for PCR amplification of the 16S rDNA gene, performed with AOB primers of CTO189f and CTO653r^26^. The CTO189f was a mixture of CTO189fA/B and CTOfC^27^. The PCR amplification was conducted in a 50-μl mixture containing 1 μl of each primer (20 μM, Takara Bio, Otsu, Japan), 0.2 μl of *Ex Taq* (5 U/μL, Takara Bio, Otsu, Japan), 5 μl of 10×*Ex Taq* buffer (Takara Bio, Otsu, Japan), 5 μl of deoxyribonucleotide triphosphate mixture (Takara Bio, Otsu, Japan), 4 μl of dNTP mixture (Takara Bio, Otsu, Japan), and 50 ng of genomic DNA. The PCR amplification steps included: initial denaturation at 94 °C for 4 min, 33 cycles at 94 °C for 1 min, annealing at 55 °C for 1 min, elongation at 72 °C for 1 min, and a final extension step at 72 °C for 10 min. PCR products of 16S rDNA genes were quantified in a 1% (wt/vol) agarose gel.

DGGE was performed using the Bio-Rad D-Code system (Bio-Rad, Richmond, CA). The PCR products of the positive AOB 16S rDNA gene were used as templates with universal primers P338f (containing a 40-bp GC clamp) and P518r to generate amplicons for DGGE. The PCR was performed using the same mixture described above. The PCR program amplification steps included: initial denaturation at 94 °C for 4 min, 30 cycles at 94 °C for 45 sec, annealing at 60 °C for 45 sec, elongation at 72 °C for 45 sec, and a final extension step at 72 °C for 10 min.

Electrophoresis tests were run using 1-mm-thick gels containing 8% polyacrylamide (for 16S rDNA PCR products). The gels were prepared using denaturing gradients ranging from 28 to 50% for the 16S rDNA fragments (100% is defined as 7 M urea and 40% vol/vol deionized formamide). These were submerged in 1×TAE buffer for 16S RNA fragments; 10 μl of the GC-clamped PCR products was added to the individual lanes on the gel. The electrophoresis of 16S rRNA PCR products were run for 5 h at a constant voltage of 150 V and a temperature of 60 °C. After electrophoresis, the gels were stained for approximately 30 min with a 5 ml 1× TAE solution containing Sybr green nucleic acid stain (Molecular Probes, Eugene, OR) in the dark and photographed with a Gel Document system (Bio-Rad, GelDoc 2000, CA). The DGGE profiles were analyzed using Quantity One software (Bio-Rad, CA).

### Band isolation, DNA sequencing, and phylogenetic analysis

Individual bands from DGGE gels were carefully excised using sterile razor blades. A Poly-Gel DNA extraction kit (Omega, USA) was used to recover DNA in the bands. The recovered DNA was further amplified using PCR with universal primers of 338f/518r. The PCR was performed in the mixture using the same method described above. The amplification procedure included an initial denaturation at 94 °C for 4 min, 30 cycles at 94 °C for 30 sec, annealing at 55 °C for 30 sec, elongation at 72 °C for 30 sec, and a final extension step at 72 °C for 10 min. The PCR products of 16S rDNA genes were quantified in a 1% (wt/vol) agarose gel, and then purified using Wizard SV Gel and PCR Clean-up System (Promega, USA). The purified PCR products were ligated to the pGEM-T Easy vector (Promega, USA) and then the plasmid was transformed into *Escherichia coli* JM109 competent cell (Takara Bio, Otsu, Japan).

The white clones were selected for sequencing analysis, carried out by a commercial company (Genewiz, Korea). The basic local alignment search tool (BLAST) was then used to compare the remaining 16S rRNA sequence in the GenBank. The accessible information was imported into MEGA 5, the gene sequences were aligned, and a phylogenetic tree was generated using performing neighbor-joining algorithms. The 16S rRNA sequences were deposited in GenBank under the following accession numbers: KF770044 to KF770091, and KF770233 to KF770241.

### FISH detection and Quantitative PCR for AOB and NOB

AOB 16s rRNA copy numbers were quantified using real-time PCR, followed documented protocols^10^. The primer set of CTO189f and CTO653r was used for AOB analysis. NOB were visualized by fluorescence *in situ* hybridization (FISH) with 16S rRNA-targeted oligonucleotide probes according to the method described by Amann *et al*.^28^. NIT3 for *Nitrobacter sp*.^29^, and NTSPA685 for the *Nitrospira mascoviensis, Nitrospira marina*, aquarium clone 710-9^30^. The images of FISH samples were captured using an Olympus-BX 61 fluorescence microscope. Because NOB was absent in the mixed halophilic culture according to FISH detection, quantitative PCR for NOB was not used in this study.

### Calculation and statistical analysis

The nitrification kinetic calculation is described in^10^. The least-squares method was used to calculate the model using Matlab 6.5. Biodiversity was calculated using Biodap software, based on the DGGE profiles. The relative intensity of the bands in the DGGE profiles over the total intensity of all detectable bands was first determined using Quantity one software (Bio-Rad, CA). These data were used for biodiversity analysis. Principal components analysis (PCA), a method using the most prominent indirect gradient analysis, was applied to the AOB community. RDA, a direct gradient analysis method, was used to further analyze the relationships between AOB community structures and reactor’s operational parameters. The statistical analysis was completed using the Conoco 4.5.

## Additional Information

**How to cite this article**: Cui, Y.-W. *et al*. The effects of salinity on nitrification using halophilic nitrifiers in a Sequencing Batch Reactor treating hypersaline wastewater. *Sci. Rep.* **6**, 24825; doi: 10.1038/srep24825 (2016).

## Supplementary Material

Supplementary Information

## Figures and Tables

**Table 1:** Diversity of bacterial community assessed using different methods at different operational phases.

	**Shannon-Wiener**	**Margalef’s**	**Simpson’s Diversity**^**a**^	**Evenness**	**Richness**
Inoculum	2.49	5.71	0.91	0.94	14
Sample d 138	2.20	5.69	0.88	0.95	10
Sample d 164	2.34	5.31	0.90	0.98	11
Sample d 185	2.14	4.91	0.88	0.97	9
Sample d 210	1.90	4.43	0.84	0.98	7
Sample d 287	2.36	4.98	0.90	0.98	11
Sample d 395	2.00	3.64	0.85	0.96	8

^a^Presence with (1-Dominance).

**Table 2:** Primary operating parameters at the seven experimental phases.

**Phase**	**Operating days**	**Salinity (g/L)**	**SRT (d)**	**VSS (mg/L)**	**Influent NH**_**4**_^**3+**^**-N**	**DO mg/L**	**SNL (mg N/(g VSS d))**	**Sample day for community analysis**
A	1–140	30 ± 1	20	3000 ± 456	48.78 ± 5.60	>3	39.02	138
B	141–165	40 ± 1	12	3405 ± 227	43.38 ± 2.96	>3	30.62	164
C	166–186	50 ± 1	12	3465 ± 183	48.32 ± 3.85	>3	33.47	185
D	187–211	60 ± 1	12	3746 ± 218	49.35 ± 1.69	>3	31.62	210
E	212–242	65 ± 1	12	3732 ± 183	54.91 ± 3.35	>3	35.31	–
F	243–288	75 ± 1	12	4181 ± 202	63.42 ± 7.83	>3	36.40	287
G	289–397	85 ± 1	12	2298 ± 238	95.47 ± 12.51	>3	99.70	395

^–^means that no sample was taken.
