# Supplementary material for: The effects of salinity on nitrification using halophilic nitrifiers in a Sequencing Batch Reactor treating hypersaline wastewater
Source: Sci Rep. 2016 Apr 25;6:24825. doi: 10.1038/srep24825 (PMC4843016; doi:10.1038/srep24825)
Supplement: Supplementary Information [file srep24825-s1.pdf]

The effects of salinity on nitrification using halophilic nitrifiers in a Sequencing Batch Reactor treating hypersaline wastewater

You-Wei Cui[1]*, Hong-Yu Zhang[1], Jie-ran Ding[1, 2], Yong-Zhen Peng[1]

1. Beijing University of Technology, College of Energy and Environmental Engineering, 100 Pingleyuan, Chaoyang District, Beijing 100124, China

2. Environmental Management College of China, 8 Road of Jingang, Beidaihe District, Qinhuangdao 066102, China

* corresponding. cyw@bjut.edu.cn

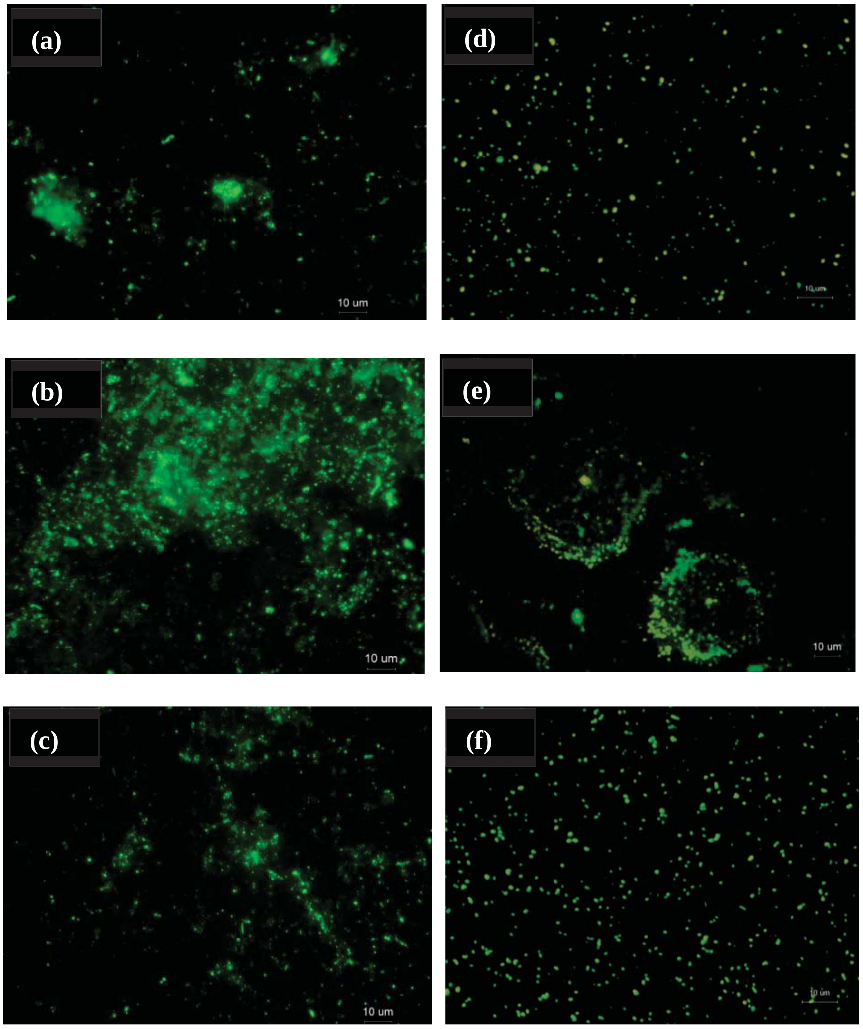

Fig. S1 FISH image of NOB detected using a probe of NIT3/EUB_{mix} and NTSPA685/ EUB_{mix}. The red color shows bacteria detected only the EUB_{mix}. The yellow color shows NOB. (a) NIT3/EUB_{mix} hybridization for the inoculated sediment, (b) NIT3/EUB_{mix} hybridization for the sludge sampled from phase A, (c) NIT3/EUB_{mix} hybridization for the sludge sampled from phase G, (d) NTSPA685/ EUB_{mix} hybridization for the inoculated sediment, (e) NTSPA685/ EUB_{mix} hybridization for the sludge sampled from phase A, (f) NTSPA685/ EUB_{mix} hybridization for the sludge sampled from phase G.

Table S1 Dice coefficients (C_s) comparing PCR-DGGE fingerprint similarities

	Inoculum	Sample at d 138	Sample d 164	Sample d 185	Sample d 210	Sample d 287	Sample d 395
Inoculum	100						
Sample d 138	42.1	100					
Sample d 164	52.1	58.3	100				
Sample d 185	53.3	59.0	94.2	100			
Sample d 210	50.1	56.9	93.4	92.3	100		
Sample d 287	45.2	42.0	70.0	68.7	66.0	100	
Sample d 395	42.2	72.9	71.5	72.3	64.4	68.9	100
